# Causal relationship between the composition of the Gut Microbiota and central precocious puberty: a two-sample Mendelian randomization study

**DOI:** 10.3389/fped.2024.1438195

**Published:** 2024-11-14

**Authors:** Minhong Chen, Xueqin Huang, Wanhong Huang, Chuangang Ding

**Affiliations:** ^1^ School of Clinical Medicine, Dali University, Dali, China; ^2^ Department of Pediatrics, Jingzhou Maternity and Child Health Hospital, Jingzhou, China; ^3^ Guangzhou Medical University, Guangzhou, China; ^4^ Department of Pediatrics, The First Affiliated Hospital of Dali University, Dali, China

**Keywords:** gut microbiota, central precocious puberty, Mendelian randomization, causal relationship, genetics

## Abstract

**Background:**

Previous observational research has demonstrated a possible association between the gut microbiota (GM) and central precocious puberty (CPP). Nevertheless, whether there is a causal relationship between the GM and CPP is uncertain due to the possibility of confounding factors influencing the result.

**Methods:**

We collected summary data from genome-wide association studies of the GM (MiBioGen, *n* = 18,340) and CPP (FinnGen Consortium, 185 case groups and 395,289 controls). Most of the participants were of European origin. Mendelian randomization analysis was utilized to investigate the causal relationship between the GM and CPP using the inverse-variance weighted average technique, the weighted median, and Mendelian randomization Egger. The reliability of the results was evaluated using the leave-one-out test and sensitivity analyses, including heterogeneity and horizontal pleiotropy testing**.**

**Results:**

According to the inverse-variance weighted average technique, there was a substantial correlation between CPP and the composition of the GM. Specifically, the relative abundance of the genus *Bacteroides* (OR 0.222, 95% CI 0.06–0.822, *P* = 0.024) and *Alistipes* (OR 0.197, 95% CI 0.056–0.697, *P* = 0.012), and others, showed significant associations. Furthermore, associations with the phylum *Euryarchaeota*, the orders *Gastranaerophilales*, and *Rhodospirillales*, the families *Bacteroidaceae*, and *Desulfovibrionaceae* were also observed. Sensitivity analyses and the leave-one-out test generated positive results for the genus *Alistipes*, implying that this genus is reliable and reduces the risk of CPP.

**Conclusions:**

The composition of the GM may have a causal effect on CPP. The present finding that *Alistipes* may be protective against CPP is expected to offer novel insights into the management of CPP.

## 1 Introduction

The term “central precocious puberty” (CPP) refers to the early onset of secondary sexual characteristics in girls and boys before the ages of 8 and 9, respectively, due to the hypothalamic-pituitary-gonadal axis (HPGA) activation (1). The global incidence of CPP has significantly increased in the last two decades (2). One in 5,000–10,000 children are affected by CPP, which is 5–10 times more common in girls than in boys, according to an epidemiologic study (3). The etiology of CPP is gradually becoming elucidated, but a definitive mechanism of development of the condition in approximately 90% of patients remains elusive, and it is therefore designated as idiopathic central precocious puberty (1, 4, 5). The early onset of puberty not only affects growth and developmental processes, but also increases the risk of developing various diseases, including depression, cardiovascular disease, obesity, and cancer (6–11). Consequently, the prevention and treatment of CPP are important for good physical and mental health.

The gut microbiota (GM), an intricate microbial ecosystem (12), is closely associated with many host diseases (13). Recent research has demonstrated that the GM produces metabolites, neurotransmitters, and neuroactive compounds that can act on the central nervous system to regulate sex hormone secretion via the gut-brain axis (14, 15). In particular, Wang *et al*. (16) showed that the intestinal microbiota and its products reverse precocious puberty in rats by inhibiting the secretion of gonadotropin-releasing hormone (GnRH) and the HPGA. This provides a new perspective regarding the pathogenesis of CPP.

According to the randomization principle, Mendelian randomization (MR) employs single nucleotide polymorphisms (SNPs) as instrumental variables (IVs) to eliminate confounding and potential reverse causation and enable an accurate evaluation of the underlying causality between exposure factors and disease risk (17–20). Utilizing data from the genome-wide association study (GWAS), we conducted a two-sample MR analysis in this study to assess the potential causal relationship between GM and CPP, and thereby aid the management of CPP.

## 2 Materials and methods

### 2.1 Study design and data sources

**Figure 1:**
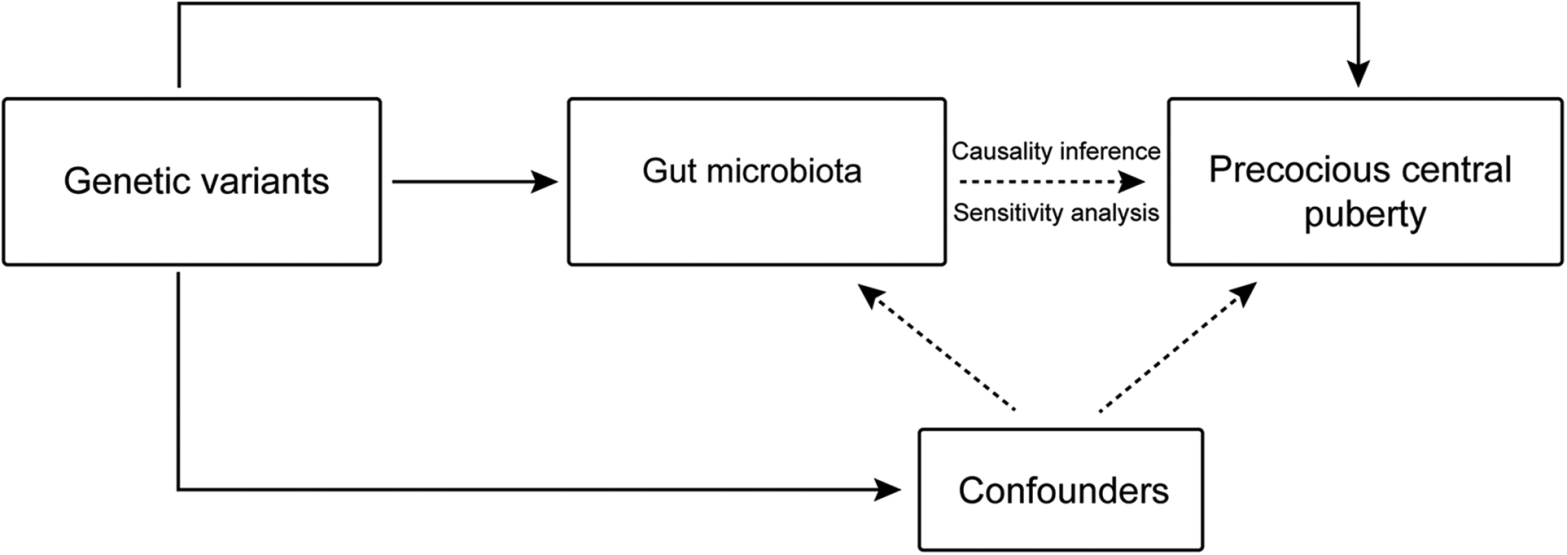
Overview of the Mendelian randomization analysis process.

Genetic variants linked to the exposure variables obtained from the GWAS summary statistics were utilized as IVs in our study, which also used GM taxa as exposure factors and CPP as the outcome factor (Figure 1). The primary analytical means of MR analysis that we employed was the inverse-variance weighted (IVW) method. Additionally, sensitivity and leave-one-out analyses were conducted to verify the reliability of the results. The MR analysis was performed having established that the following conditions were satisfied: (i) the IV and exposure had a strong correlation; (ii) the IV was unrelated to any confounding factors; and (iii) the IV only influenced the outcome through the target exposure.

The GWAS data for the GM came from the MiBioGen study, which assembled the 16S rRNA gene sequencing profiles of 18,340 individuals (13,266 from European populations) and included 211 units (comprising families, phyla, orders, genera, and classes) (21). These data were accessed from the study website (www.mibiogen.org). The GWAS data for CPP were sourced from the FinnGen Consortium R10 release (https://r10.finngen.fi/), which included 185 case groups and 395,289 controls overall (22). All of the study's participants were from Europe.

### 2.2 Data extraction and filtering

#### 2.2.1 Selection of instrumental variables

SNPs in the genes representing the exposures that were extracted from original GWAS meta-analyses or public databases were used as the instrumental variables (IVs) for the exposure factors. A threshold for genome-wide significance (*P* < 1 ×  10^−5^) was established in order to achieve a thorough comprehension of the exposure to genetic variation. Concurrently, to ensure the independence of the IVs and mitigate linkage disequilibrium, we stipulated an r^2^ < 0.01, and a clumping window size of 500 kb (23). Finally, we discarded SNPs with an F statistic < 10 to ensure only close associations between exposure factors and IV would be obtained. Equation 1 is the formula used to calculate the F statistic, where R^2^ is the percentage of exposure variance explained by a particular SNP and N is the sample size (24). R^2^ was determined using the Equation 2.


(1) $$F=R^{2}\;\times \;(N\;-\;2)/(1\;-\;R^{2})$$

(2) $$R^{2}\;=\;(2\;\times \;{\mathrm{Beta}}^{2})/(2\;\times \;{\mathrm{Beta}}^{2}\;+\;2\;\times \;{\mathrm{SE}}^{2\;}\times \;N)$$

#### 2.2.2 Outcome data extraction and filtering

From the original GWAS meta-analyses, SNPs connected with relevant diseases were taken out and used as IVs for the outcome variables. SNPs that exhibited a close association with the relevant exposure diseases and were also present in the outcome variables were then screened.

### 2.3 Construction of the relationships between instrumental variables, exposures, and outcomes

The harmonization of selected IVs with outcome-associated SNPs was used to eliminate palindromic sequences and allele-incompatible SNPs. Furthermore, to accept the assumption of exclusivity for this MR study, we required an outcome-related SNP significance level of *P* > 1 ×  10 ^−5^. Anomalous outliers were removed using the MR_radial. Li *et al*. (25) demonstrated that CPP is associated with obesity, the consumption of beverages and sweets, and sleep quality. Therefore, to remove the influence of these confounding factors, we conducted a systematic search of the PhenoScanner database (http://www.phenoscanner.medschl.cam.ac.uk/) to remove the associated SNPs.

### 2.4 MR analysis

We used the weighted median (WM) and MR–Egger as auxiliary methods in addition to the IVW method as the primary strategy for the MR analysis. When using the IVW approach, it is assumed that each IV is valid and does not exhibit pleiotropy. However, this approach does not take into account the presence of an intercept, which resists bias and has the highest statistical efficacy (26). The MR–Egger method considers the intercept and is susceptible to the effect of anomalous IVs, but can generate unbiased estimates, even using invalid IVs (27). It has been established that the WM can be calculated using a considerable proportion of invalid IVs (approximately 50%), and generates a higher causal efficacy and lower error than the MR–Egger (28). Finally, a stable causal relationship between exposures and outcomes was only considered if *P* < 0.05 for the IVW.

### 2.5 Sensitivity analysis

Sensitivity analyses, such as heterogeneity and horizontal pleiotropy tests, were performed to evaluate the robustness of the primary findings. The heterogeneity of the SNPs was evaluated using Cochran's *Q*-test, and the horizontal pleiotropy was evaluated using the MR–Egger intercept. When *P* > 0.05, the lack of heterogeneity or pleiotropy was acknowledged. Additionally, each SNP's impact on causation was assessed using the leave-one-out test (29).

We used the “TwoSampleMR” package in the R software environment (version 4.3.2) to perform these analyses.

## 3 Results

We obtained 2,641 eligible SNPs and 196 gut microbial taxa using the described screening criteria for IVs, performing allelic consistency testing, and removing confounders. The F-statistic values for these SNPs were all >10, indicating a stable statistical effect among the selected IVs (Supplementary File S1, Table S1).

We performed MR analysis using three methods (WM, IVW, and MR–Egger), with the results of the IVW method serving as the primary index. The detailed findings are shown in Supplementary File S1, Table S2. Based on the IVW analysis, 10 gut bacterial taxa had *P*-values below the threshold of <0.05 and were therefore included in the subsequent analysis (Table 1). We used MR_radial to remove the outliers and after repeated causal analyses found that one phylum (IVW: OR 0.536, 95% CI 0.31–0.926, *P* = 0.025 for *Euryarchaeota*), two orders (IVW: OR 0.446, 95% CI 0.202–0.987, *P* = 0.046 for *Gastranaerophilales*; OR 2.079, 95% CI 1.003–4.309, *P* = 0.049 for *Rhodospirillales*), families (IVW: OR 0.222, 95% CI 0.06–0.822, *P* = 0.024 for *Bacteroidaceae*; OR 0.250, 95% CI 0.07–0.900, *P* = 0.034 for *Desulfovibrionaceae*), and genus (IVW: OR 0.222, 95% CI 0.06–0.822, *P* = 0.024 for *Bacteroides*; OR 0.197, 95% CI 0.056–0.697, *P* = 0.012 for *Alistipes*) were significantly associated with CPP (Table 1).

**Table 1:** Mendelian randomization results for Gut microbiota and central precocious puberty.

Exposure	Nsnp	IVW		Weighted median		MR Egger	
		OR (95%CI)	pval	OR (95%CI)	pval	OR (95%CI)	pval
phylum.Euryarchaeota.id.55	12	0.536 (0.31,0.926)	**0** **.** **025**	0.562 (0.273,1.155)	0.117	0.321 (0.028,3.667)	0.382
class.Betaproteobacteria.id.2867	8	5.725 (0.957,34.266)	0.056	6.132 (0.844,44.522)	0.073	0.034 (0.001,2.074)	0.158
order.Gastranaerophilales.id.1591	9	0.446 (0.202,0.987)	**0**.**046**	0.439 (0.162,1.189)	0.105	0.734 (0.069,7.842)	0.806
order.Rhodospirillales.id.2667	14	2.079 (1.003,4.309)	**0**.**049**	2.102 (0.809,5.458)	0.127	0.587 (0.031,11.115)	0.728
order.Burkholderiales.id.2874	8	2.984 (0.43,20.701)	0.152	2.984 (0.430,20.701)	0.269	2.921 (0.673,12.676)	0.225
family.Bacteroidaceae.id.917	9	0.222 (0.06,0.822)	**0**.**024**	0.256 (0.054,1.207)	0.085	1.451 (0.002,1,274.04)	0.917
family.Desulfovibrionaceae.id.3169	9	0.250 (0.07,0.900)	**0**.**034**	0.231 (0.044,1.200)	0.081	0.428 (0.018,10.179)	0.616
genus.Bacteroides.id.918	9	0.222 (0.060,0.822)	**0**.**024**	0.256 (0.049,1.342)	0.107	1.451 (0.002,1,274.04)	0.917
genus.Alistipes.id.968	12	0.197 (0.056,0.697)	**0**.**012**	0.210 (0.042,1.061)	0.059	4.834 (0.014,1,689.232)	0.609
genus.Ruminococcusgauvreauiigroup.id.11342	11	0.346 (0.115,1.042)	0.059	0.422 (0.106,1.680)	0.221	1.251 (0.014,113.702)	0.925

Bold values indicate the significance threshold p < 0.05 for IVW, corresponding to a significant causal relationship between gut bacteria and central precocious puberty.

**Figure 2:**
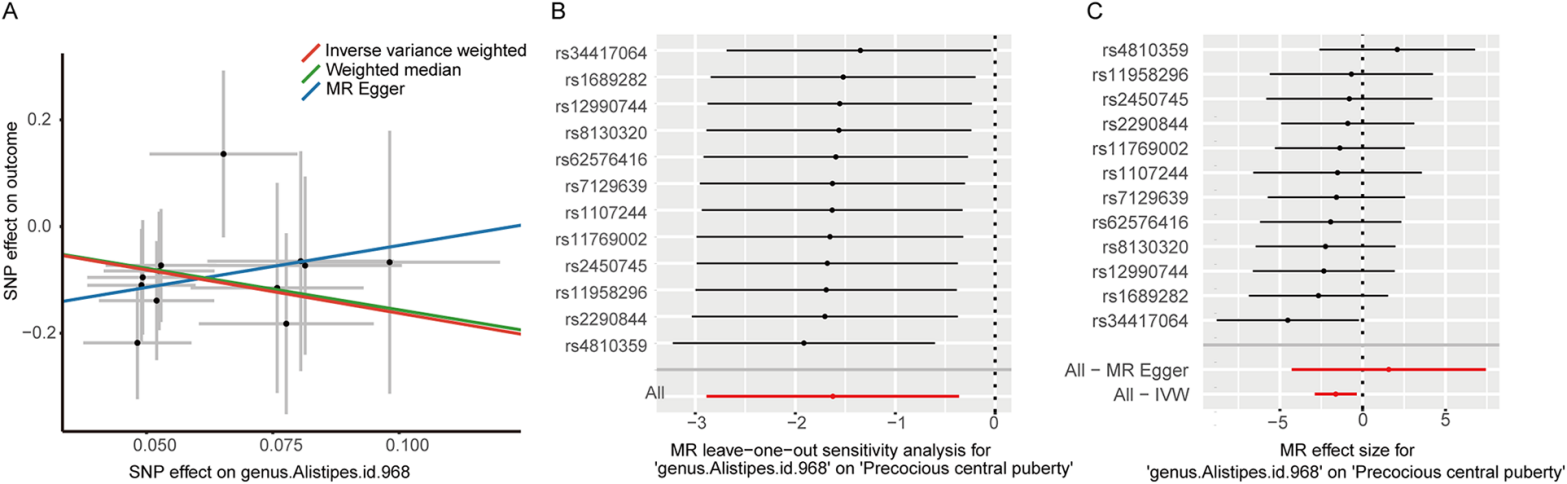
**(A)** Scatter plots illustrating the causal effect of genus Alistipes on central precocious puberty; **(B)** Leave-one-out analysis for genus Alistipes on central precocious puberty; **(C)** MR effect size for genus Alistipes on central precocious puberty.

Finally, in conjunction with the IVW results, we performed leave-one-out testing, and only obtained a positive result for the genus *Alistipes* (OR 0.197, 95% CI 0.056–0.697, *P* = 0.012), implying that this finding was stable and that it is protective against CPP (Figure 2). It should be noted that, because MR–Egger does not necessitate a forced regression to the origin and exhibits limited statistical efficacy, it is acceptable to present findings that are in the direction opposite to that obtained using the IVW and WM methods. After having performed leave-one-out testing, an additional six gut bacterial taxa were excluded, owing to their influence on single SNPs, which resulted in unstable results. Additional information is provided in Supplementary Figure 1. Notably, we did not detect weak instrumental bias (F > 10), horizontal pleiotropy (MR–Egger intercept *P* > 0.05), or heterogeneity (Cochran's *Q*-test *P* > 0.05) (Table 2).

**Table 2:** Sensitivity analysis results for Gut microbiota and precocious central puberty.

Exposure	Outcome	Heterogeneity		Pleiotropy	
		Q	Q_pval	intercept	intercept_pval
genus.Alistipes.id.968	Precocious central puberty	4.992	0.932	−0.193	0.298

## 4 Discussion

In this research, we novelly conducted a two-sample MR study to determine the specific causal effects of 196 gut microbial taxa on accelerating or inhibiting the development of CPP. The causal evaluations were examined and verified through multiple analyses, including IVW, WM, MR-Egger, sensitivity, and leave-one-out tests. IVW estimates revealed that the phylum *Euryarchaeota*, the order Candidatus *Gastranaerophilales*, the family *Bacteroidaceae*, the family *Desulfovibrionaceae*, the genus *Bacteroides*, and the genus *Alistipes* could potentially reduce the risk of CPP while the order *Rhodospirillales* might enhance the risk of CPP. Eventually, after sensitivity analyses and the leave-one-out test, the genus *Alistipes* was suggested to stably prevent the occurrence of CPP causally. Our findings elucidate whether the human GM can participate in the pathogenesis of CPP and which gut microbial taxon can alter the risk of CPP.

Driven by the early activation of HPGA, the initiation of CPP is highly related to disorders in the endocrine system, especially abnormal sexual maturation and secretion of sex hormones (30). Meanwhile, GM has been demonstrated to play an important role in the disruption of the hormone system and the progression of a series of systemic diseases (31). Since the composition of GM is significantly different in different puberty stages, the effect of GM during the physiological pubertal development may further indicate the potential connection between GM and pathological puberty (32). With the proposal of the sex hormone-gut microbiome axis, increasing studies have focused on the role of GM on pubertal disorders, especially precocious puberty, and the underlying mechanisms (33). A study involving 91 CPP patients investigated the alteration of the composition of GM in CPP through bioinformatics and suggested that the genus *Streptococcus* could act as a marker for CPP (34). Another observational research identified GM dysbiosis within 25 CPP patients and altered gut microbial taxa in CPP were similar to those in obesity, while obesity had already been recognized as the main cause for CPP (35, 36). In addition, most enriched gut genera in CPP were defined as short-chain fatty acids (SCFAs)-producing bacteria. At the same time, SCFAs can elevate the expression of metabolic peptides from adipocytes and are essential for obesity-induced precocious puberty (16). However, limited populations as well as potential reverse causality and confounders in observational studies hinder the exploration of the causal effects of GM on the risk of CPP. Although existing research has indicated that GM might be correlated to CPP, whether the links in phenotype were solely clinically manifested, achieved through obesity, or causal remains unknown. Therefore, we carried out MR research based on the large-scale GWAS data that can successfully avoid reverse causalities and confounding factors to investigate the causal associations directly between GM and CPP. As a result, we identified the causal impacts of GM on the risk of CPP, which not only was consistent in previous observational studies but also provided genetic evidence for their strong causal links. In terms of mechanisms underlying the causalities, according to a narrative review, GM can produce several metabolically active substances, together with GM, both have been shown to influence sex hormone secretion through nutritional status, hormone regulation, and metabolic pathways (37). On the one hand, GM can directly alter the level of estrogens and androgens in the host organism (38). On the other hand, as typical GM metabolites, SCFAs (31) (mainly consisting of acetate, propionate, and butyrate), neurotransmitters (14) (e.g., serotonin and dopamine), and neuroactive compounds (34) (such as nitric oxide), are important mediators in the gut-brain axis and the transduction of sex hormone signaling, probably influencing the progression of CPP.

Within positive IVW estimates, *Bacteroides* was found to be reduced in individuals with obesity by several animal and human studies (39–41). The genus *Bacteroides* principally produces propionate, which is anti-lipogenic (42) and anti-inflammatory (43), enhancing satiety (44), and ameliorating insulin resistance (45). The negative links between the abundance of *Bacteroides* with obesity might support our results that the family *Bacteroidaceae* and the genus *Bacteroides* could inhibit the occurrence of CPP. Previous studies also showed that propionate and butyrate activated the HPGA by increasing leptin gene expression (46). As for the family *Desulfovibrionaceae*, an observational study showed that the abundance of *Desulfovibrio* in the feces of obese mice was positively correlated with the circulating concentration of GnRH (15), which might promote intestinal inflammation and sexual development (47, 48). Our MR research further identified the impact of the family *Desulfovibrionaceae* in causally decreasing the risk of CPP. Additionally, as a CPP promotion taxa suggested by our MR research, the order *Rhodospirillales* is recognized as a proinflammatory factor, and its metabolites such as lipopolysaccharide could participate in inflammation and immune responses in hosts (49). Furthermore, the order *Rhodospirillales* is also associated with amino acids, nitrogen, vitamins, and cofactor metabolisms, which might affect the production of sex hormones and the development of CPP (50).

To be noted, the genus *Alistipes* was the exclusive positive taxa causally related to the risk of CPP after being verified via the leave-one-out test. *Alistipes* is a recently discovered genus of anaerobic bacteria in the healthy human gut (51) that produces SCFAs (52). Regarding the abundance of *Alistipes* in patients with CPP, a previous observational study showed that this genus was significantly more abundant in patients with CPP groups than in healthy controls or individuals with over-weight (14). However, another study performed in humans showed no significant difference in the abundance of *Alistipes* between patients and healthy controls (31). The outcomes of these studies were not consistent, which may be attributable to discrepancies in ethnicity, species, host metabolic status, and/or measurement methods. Our MR research extensively minimized bias and elucidated the protective role of the genus *Alistipes* for CPP, providing a potential treatment biomarker for CPP. An animal study demonstrated that the addition of acetate, propionate, butyrate, or a combination of these to a high-fat diet reduces GnRH release and reverses precocious puberty (16). Other scholars proposed that *Alistipes* transplantation could be used to alleviate high-fat diet-induced obesity and its associated complications (53), which also supported our causal estimates. In terms of the underlying mechanism, acetate produced by *Alistipes* has been reported to regulate lipid metabolism (54) and appetite (55). Butyrate can protect the intestinal mucosa (56) and reduce appetite (57) and the concentrations of inflammatory mediators (58), such as nitric oxide, which acts as a neurotransmitter and promotes the production of sex hormones (59). Furthermore, butyrate is beneficial for mental health (60) and psychological issues have been identified as a significant regulator of sex hormone production (61). However, further research is still required to elucidate the effects of differing abundances of gut microbial taxa and the mechanism of the effects of their metabolites in patients with CPP.

In the present study, we used genetic variables to determine whether there is a causal relationship between exposure factors and the disease of interest, thereby minimizing the effects of confounding factors. Nevertheless, it is important to acknowledge the limitations of the study. The participants in the study were predominantly of European origin, and therefore the findings may not be applicable to individuals of other ethnic origins. Furthermore, the abundances and effects of gut microbial taxa are influenced by several factors, including age, sex, and dietary habits. However, we did not perform subgroup analyses in the present study.

## 5 Conclusion

In the present study, we have shown that *Alistipes* may be protective against CPP. This finding provides novel information to aid the management of CPP. Nevertheless, further large-scale animal and human studies are required to elucidate the mechanism whereby the composition of the GM affects CPP and to develop related strategies for the prevention and treatment of CPP.

## Data Availability

The original contributions presented in the study are included in the article/Supplementary Material, further inquiries can be directed to the corresponding author.

## Supplementary material

Supplementary Figure 1 Leave-one-out sensitivity analysis for phylum Euryarchaeota, order Gastranaerophilales, order Rhodospirillales, family Bacteroidaceae, family Desulfovibrionaceae, and genus Bacteroides on central precocious puberty.
