# Supplementary figures and images for: Causal relationship between the composition of the Gut Microbiota and central precocious puberty: a two-sample Mendelian randomization study
Source: Front Pediatr. 2024 Nov 14;12:1438195. doi: 10.3389/fped.2024.1438195 (PMC11609932; doi:10.3389/fped.2024.1438195)

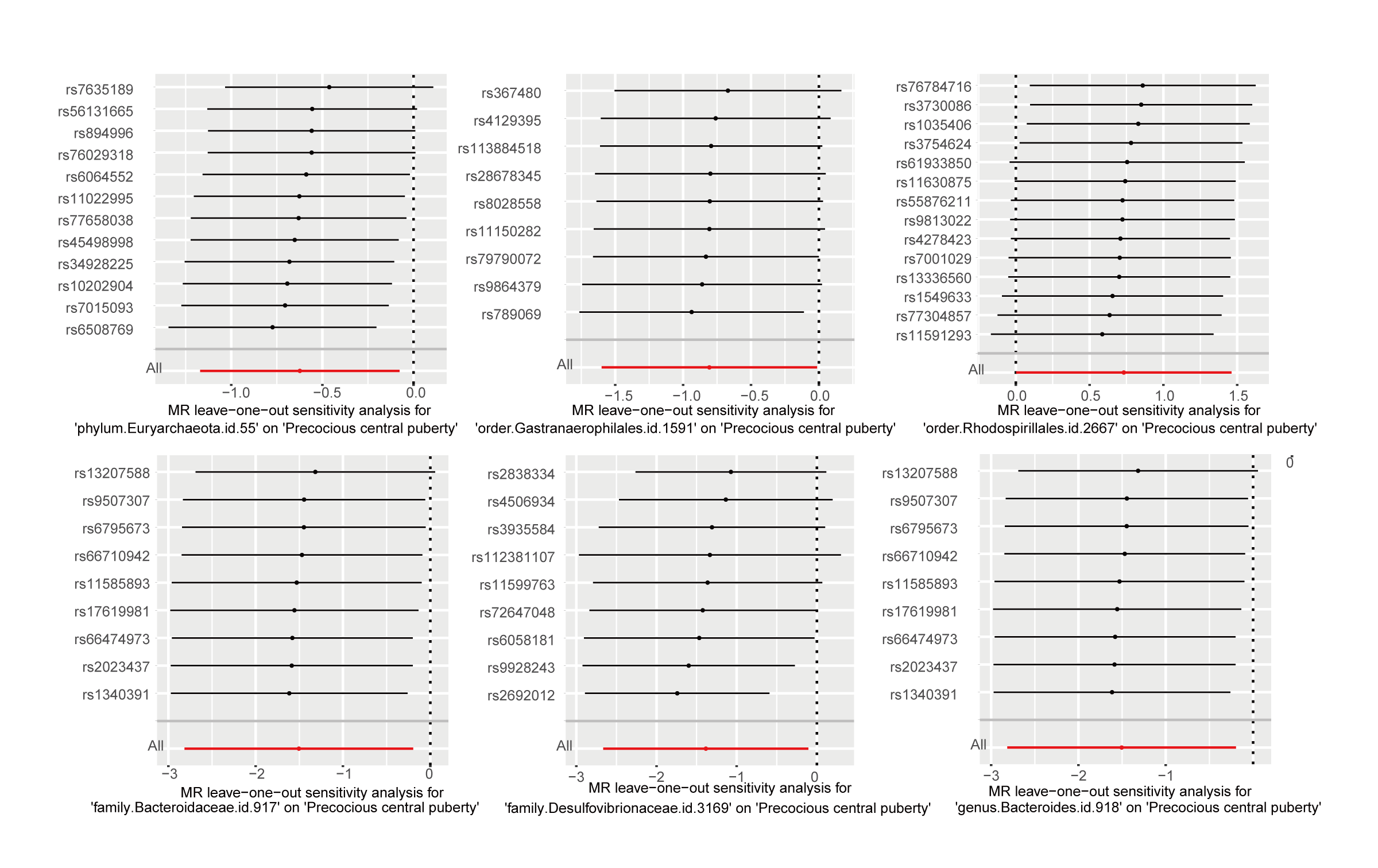

Supplement: Supplementary Figure 1 — Leave-one-out sensitivity analysis for phylum Euryarchaeota, order Gastranaerophilales, order Rhodospirillales, family Bacteroidaceae, family Desulfovibrionaceae, and genus Bacteroides on central precocious puberty. [file Image1.tif]
